# Heparin-binding protein is important for vascular leak in sepsis

**DOI:** 10.1186/s40635-016-0104-3

**Published:** 2016-10-04

**Authors:** Peter Bentzer, Jane Fisher, HyeJin Julia Kong, Mattias Mörgelin, John H. Boyd, Keith R. Walley, James A. Russell, Adam Linder

**Affiliations:** 1 Department of Anesthesia and Intensive Care, Helsingborg Hospital, Helsingborg, Sweden; 2 Department of Clinical Sciences Lund, Lund University, Lund, Sweden; 3 Department of Infectious Diseases, University of Lund and Skåne University Hospital, Getingevägen, Lund SE-221 85 Sweden; 4 Centre for Heart Lung Innovation, Division of Critical Care Medicine, St. Paul’s Hospital, University of British Columbia, Vancouver, BC Canada

**Keywords:** Septic shock, Heparin-binding protein (HBP), Vascular leak, Acute respiratory distress syndrome, Permeability

## Abstract

**Background:**

Elevated plasma levels of heparin-binding protein (HBP) are associated with risk of organ dysfunction and mortality in sepsis, but little is known about causality and mechanisms of action of HBP. The objective of the present study was to test the hypothesis that HBP is a key mediator of the increased endothelial permeability observed in sepsis and to test potential treatments that inhibit HBP-induced increases in permeability.

**Methods:**

Association between HBP at admission with clinical signs of increased permeability was investigated in 341 patients with septic shock. Mechanisms of action and potential treatment strategies were investigated in cultured human endothelial cells and in mice.

**Results:**

Following adjustment for comorbidities and Acute Physiology and Chronic Health Evaluation (APACHE) II, plasma HBP concentrations were weakly associated with fluid overload during the first 4 days of septic shock and the degree of hypoxemia (PaO_2_/FiO_2_) as measures of increased systemic and lung permeability, respectively. In mice, intravenous injection of recombinant human HBP induced a lung injury similar to that observed after lipopolysaccharide injection. HBP increased permeability of vascular endothelial cell monolayers in vitro, and enzymatic removal of luminal cell surface glycosaminoglycans (GAGs) using heparinase III and chondroitinase ABC abolished this effect. Similarly, unfractionated heparins and low molecular weight heparins counteracted permeability increased by HBP in vitro. Intracellular, selective inhibition of protein kinase C (PKC) and Rho-kinase pathways reversed HBP-mediated permeability effects.

**Conclusions:**

HBP is a potential mediator of sepsis-induced acute lung injury through enhanced endothelial permeability. HBP increases permeability through an interaction with luminal GAGs and activation of the PKC and Rho-kinase pathways. Heparins are potential inhibitors of HBP-induced increases in permeability.

**Electronic supplementary material:**

The online version of this article (doi:10.1186/s40635-016-0104-3) contains supplementary material, which is available to authorized users.

## Background

A key feature in the pathophysiology of adult respiratory distress syndrome (ARDS) and septic shock is increased microvascular permeability; however, the current understanding of the underlying mechanisms is limited [1]. Furthermore, no therapy directly targeting increased microvascular permeability is currently available in sepsis.

Heparin-binding protein (HBP), also known as azurocidin or CAP37, is stored in secretory vesicles and azurophilic granules of neutrophils and is released early upon neutrophil adhesion and during neutrophil extravasation. Bacterial products induce release of HBP leading to increased vascular leakage by acting on endothelial cells through largely unknown mechanisms [2]. It has been shown that HBP binds to cell surface proteoglycans, but the importance of this binding for permeability increases has not been investigated [3, 4]. Moreover, the importance of HBP on the pathophysiology and outcomes of sepsis is unclear. Support for the importance of the permeability-increasing effect of HBP may be inferred from the observation that elevated plasma levels of HBP are associated with shock in septic patients [5–7]. Furthermore, while patients with severe ARDS have been shown to have higher HBP levels than those with less severe ARDS [8], it is unclear if increased levels of HBP merely reflect injury severity or if there is a causal relationship between HBP and ARDS.

Based on these considerations, the first objective of the present study was to test the hypothesis that elevated levels of HBP are associated with severity of fluid overload and ARDS—as indirect markers of increased permeability in a cohort of patients with septic shock. Based on findings compatible with a role for HBP in vascular leakage in septic shock, our second objective was to use in vitro and in vivo models to investigate the cellular mechanisms involved in the permeability-increasing effect of HBP and, finally, to explore the potential for heparins to mitigate HBP-induced increased permeability.

## Methods

### Human studies

#### Patient population

Plasma concentrations of HBP were analyzed in a subgroup of patients included in the Vasopressin and Septic Shock Trial (VASST) cohort of septic shock patients [9]. VASST was a multicenter randomized double-blind controlled trial in which adult patients with septic shock requiring vasopressor support (at least 5 μg/min of noradrenaline) for at least 6 h despite adequate fluid resuscitation were eligible for inclusion (*n* = 778). Patients were randomized to receive either masked vasopressin or noradrenaline to reach a target mean arterial pressure of 65 to 75 mmHg until shock had resolved. Infusion of the study drug was started 12 ± 9 h after meeting inclusion criteria and plasma that was collected at baseline (within 2 h of start of infusion of the study drug) was available for 341 patients. The research ethics boards of all 27 participating centers approved the VASST (Current Controlled Trials number, ISRCTN94845869). Written informed consent was obtained from patients, next of kin, or surrogate decision-makers as appropriate. Plasma concentration of HBP was measured in duplicate blinded to clinical outcomes using a commercial HBP ELISA (Axis-Shield Diagnostics Ltd.). Plasma concentration of IL-6 was measured in duplicate using a Luminex multiplex bead assay (Luminex, Austin, TX).

#### Outcome measures

The primary outcome was the relationship between HBP concentration in plasma and percent fluid overload. Percent fluid overload is a marker of increased vascular leakage and was calculated at 6, 12, 24, and 48 h after inclusion using the following formula: (fluid intake − fluid output)/(body weight (kg))*100 [10, 11]. The secondary outcome was the association between plasma HBP concentration at baseline and lung permeability. Because radiologic data and ventilator settings were unavailable, PaO_2_/FiO_2_ was used as a surrogate marker of increased lung permeability and ARDS. In addition, we examined the association between plasma HBP concentration at baseline and severity of shock measured as norepinephrine dose and plasma lactate concentration during the first 5 days after admission.

### In vitro studies

#### Human endothelial cell model

Immortalized human umbilical vein endothelial cells (EA.hy926, American Type Culture Collection) were cultured in Dulbecco’s modified Eagle’s medium (DMEM) supplemented with 10 % fetal bovine serum (FBS) (both from Life Technologies). For all permeability measurements, cells were cultured to confluence (3 days) on ThinCert transparent inserts with a 3-μm pore size (Greiner Bio-One). Experiments with these cells were carried out in serum-free DMEM. Human recombinant HBP was used for all in vitro experiments (R&D Systems) and in vivo experiments (Novoprotein).

#### Permeability assays

Trans-endothelial electrical resistance (TEER) reflects paracellular small molecule permeability, and a decrease in TEER reflects an increased permeability. TEER was measured using an EVOM system with STX2 electrodes (World Precision Instruments) as described previously [12]. Confluence of the monolayer was confirmed by microscopic inspection and by measuring a resistance across the monolayer of at least 50 Ω more than that of a well with no cells. To validate that changes in TEER reflected physiologically important permeability changes, macromolecule permeability was determined by measuring diffusion of streptavidin-conjugated horseradish peroxidase (HRP) (MW ≈ 100 kDa) in some of the experiments as described in detail in Additional file 1.

#### Glycosaminoglycan digestion

Cells cultured on permeable inserts were treated with 15 mU/mL of Heparinase III (New England Biolabs) or 2.5 mU/mL Chondroitinase ABC (R&D Systems) for 1 h at 37 °C [13]. Cells were then stimulated with HBP and TEER, and HRP passage was measured after 1.5 h.

#### Evaluation of HBP signaling pathways

For inhibition of signaling pathways, cells were pretreated for 1 h at 37 °C with Y-27 632 (Tocris) at a concentration of 1 μM [14] or Calphostin C (Tocris) at a dose of 50 nM [15] to inhibit Rho-kinase and protein kinase C (PKC), respectively.

#### Effects of heparin compounds on HBP-induced increased permeability

The putative inhibitors of HBP were unfractionated heparin (UFH) (Leo) and three low molecular weight heparins (LMWHs): dalteparin (Pfizer), enoxaparin (Sanofi-Aventis), and tinzaparin (Leo). Varying doses of UFH were tested to determine the minimally effective dose. Therapeutic plasma levels of heparin in humans are 0.3–0.7 U/mL, and a concentration range of 0.01–100 U/mL was examined [16]. The LMWHs were dosed to obtain the clinical therapeutic level of 1 anti-Xa IE/mL [17]. HBP (10 μg/mL) was mixed with the various heparins, incubated at 37 °C for 20 min, and cells were subsequently stimulated with this mixture.

### In vivo studies

Lund University Ethical Committee for Animal Research approved the experimental protocol. Adult male C57BL/6 mice (Taconic) weighing 26 ± 2 g were used. Mice were treated in accordance with the National Institutes of Health for the Care and Use for Laboratory Animals. After induction of anesthesia and preparation, as described in detail in Additional file 1, animals received one of three treatments: (1) HBP 100 μg intravenously followed by an infusion of 2.5 μg/g/h for 1 h (*n* = 3), (2) UFH at a dose of 0.4 U/g followed by HBP 100 μg intravenously followed by infusion of HBP as described above (*n* = 3). This dose has previously been shown to double activated partial thromboplastin time in mice and is therefore in the therapeutic range [18]. (3) Control: bolus dose of 100 μL of vehicle (10 mM phosphate-buffered saline (PBS)) followed by infusion of vehicle at 1.25 μL/g/h (*n* = 3).

At 1 h after start of treatment, animals were killed by exsanguination and the lungs were collected for electron microscopy and histologic analysis as described in Additional file 1. An investigator blinded to the treatment status of the animals performed preparation and analysis of electron microscopic and histologic images as described in detail previously [19]. Briefly, histologic analysis was done by scoring three lung sections from each animal for alveolar thickness, capillary congestion, and cellularity using a score from 0 to 3 with 3 being the highest injury score. An overall score was calculated by averaging the three indices of injury. The shed blood was collected for measurement of plasma concentrations of HBP as described above. Electron microscopy and histology preparations were compared to those obtained from mice sacrificed at 4 h after intraperitoneal injection of lipopolysaccharide (LPS) from *Escherichia coli* 0111:B4 (Sigma-Aldrich) in a dose of 0.25 mg [20] after preparation as described above.

#### Statistical analysis

Comparisons between groups were made using the non-parametric Mann-Whitney test, Student’s *t* test, one-way ANOVA, and two-way repeated measures ANOVA as appropriate. Spearman’s non-parametric correlation coefficient (rho) was used to assess correlations between HBP levels and percent fluid overload and PaO_2_/FiO_2_. Two-tailed *P* values of less than 0.05 were considered to be significant. Adjusted analyses were done by a logistic regression model for presence of severe ARDS (PaO_2_/FiO_2_ ≤100 mmHg [≤13.3 kPa]) and adjusting for age, gender, Acute Physiology and Chronic Health Evaluation (APACHE) II, comorbidities, and physiological parameters and laboratory variables that differed significantly between the patients with PaO_2_/FiO_2_ ≤100 or >100 mmHg, respectively (Table 1). Data are expressed as mean ± standard deviation unless stated otherwise. Data were analyzed using GraphPad Prism (version 6.0, GraphPad Software, Inc.) and SPSS (version 19.0).

**Table 1:** Patient characteristics at baseline

	All patients (*n* = 341)	PaO_2_/FiO_2_ >100 (*n* = 283)	PaO_2_/FiO_2_ ≤100 (*n* = 53)	*P* value
Male, *n* (%)	201 (59)	163 (57)	37 (70)	0.13
Age, years (median (IQR))	63 (50.6–72.4)	63 (50.6–72.2)	64 (49.8–73.6)	0.93
Caucasian, *n* (%)	307 (90)	261 (92)	42 (79)	<0.01
APACHE II (median (IQR))	26 (21–32)	26 (21–32)	29 (24–35)	<0.01
Comorbidities, *n* (%)				
Chronic heart failure	26 (8)	21 (7.4)	5 (9.4)	0.82
COPD	58 (17)	53 (19)	5 (9.4)	0.15
Chronic steroids	72 (21)	60 (21)	11 (21)	1.0
Chronic dialysis	30 (9)	23 (8.1)	6 (11)	0.62
Chronic hepatic failure	37 (11)	29 (10)	8 (15)	0.43
Infection site, *n* (%)				
Lung	147 (43)	112 (40)	34 (64)	<0.01
Abdomen	89 (26)	80 (28)	8 (15)	0.07
Other	105 (31)	91 (32)	11 (21)	0.14
Physiological and laboratory variables at baseline, median (IQR)				
MAP (mmHg)	56 (50–62)	56 (50–62)	55 (48–61)	0.22
Lactate (mmol/L)	1.7 (0.9–3.4)	1.6 (0.8–3.2)	2.5 (1.3–5.0)	<0.01
Norepinephrine (μg/min)	13 (8–25)	12 (8–22)	23 (11–38)	<0.01
WBC (10^9^ cells/L)	14 (8–21)	14 (8–21)	11 (7–19)	0.18
Platelets	172 (90–259)	174 (90–268)	153 (108–238)	0.25
Temperature (°C)	38.6 (37.7–39.3)	38.5 (37.7–39.3)	38.7 (37.8–39.2)	0.49
PaO_2_/FiO_2_	192 (142–260)	205.3 (162–271)	99 (82.5–110.8)	<0.01
IL-6 (pM)	4.3 (1.5–37)	5.6 (1.5–24)	18.9 (2.1–422)	<0.01
Outcomes other than mortality, median (IQR)				
DAF ventilator support	9 (0–21)	13 (1–22)	0 (0–2)	<0.01
DAF renal replacement therapy	27 (7–28)	28 (12–28)	4 (1–16)	<0.01

Groups were compared using the Student’s *t* test, or Mann-Whitney test, or chi-squared test as appropriate

*DAF* days alive and free, *WBC* white blood cell count, *PaO*_*2*_ arterial partial pressure of oxygen, *FiO*_*2*_ fraction of inspired oxygen

## Results

### Plasma HBP is associated with fluid overload

**Fig. 1:**
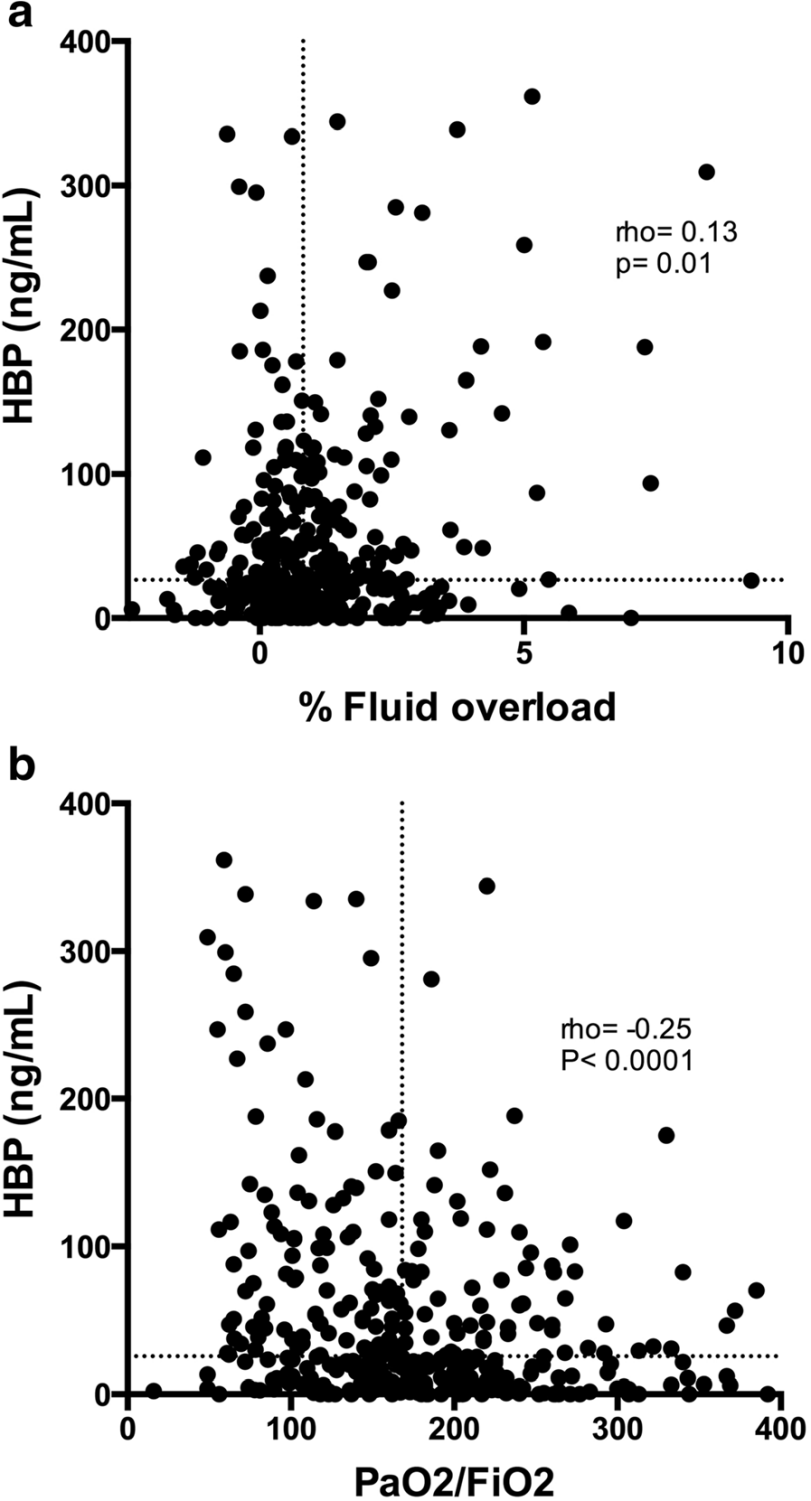
Elevated plasma HBP levels are associated with markers of increased vascular leakage. **a** Scatterplot of plasma HBP levels at baseline and percent fluid overload at 6 h after admission. *Dotted lines* mark median value for HBP and percent fluid overload, respectively. **b** Scatterplot of plasma HBP levels at baseline and lowest PaO_2_/FiO_2_ during the first 5 days after admission. *Dotted lines* mark median value for HBP and PaO_2_/FiO_2_ fluid overload, respectively. Spearman’s non-parametric correlation coefficient (rho) is given in the figures

Median plasma concentration of HBP at baseline for the whole cohort was 25 ng/mL (range, 0–361, interquartile range (IQR) 8–71). For comparison, median HBP levels are reported to be 6 (range, 2–9 ng/mL) in healthy controls using a similar methodology [21]. We tested the hypothesis that increased vascular leakage, as reflected by percent fluid overload, was correlated with HBP concentration. Increased plasma HBP was very weakly correlated with percent fluid overload at 6 h (rho 0.13, *P* = 0.01, Fig. 1a).

### HBP is associated with severity of hypoxemia

Increased plasma concentration of HBP correlated weakly with the lowest PaO_2_/FiO_2_ at any time in the first 5 days after admission, as an indicator of severity of ARDS (rho −0.25, *P* < 0.001, Fig. 1b). Patients with severe hypoxemia as defined by a PaO_2_/FiO_2_ ≤100 mmHg at any time in the first 5 days after admission were hemodynamically more compromised at baseline and had higher APACHE scores than patients with PaO_2_/FiO_2_ >100 mmHg (Table 1). Plasma HBP concentration was 47 (IQR 23–123) and 22 (IQR 7–62) in the groups with PaO_2_/FiO_2_ ≤100 mmHg and PaO_2_/FiO_2_ >100 mmHg, respectively (*P* < 0.01). Logistic regression adjusting for ethnicity, gender, age, APACHE II, site of infection, chronic heart failure, COPD, chronic steroid treatment, chronic dialysis and chronic hepatic failure, lactate concentration, norepinephrine dose and IL-6 concentrations showed that plasma HBP concentrations remained associated with presence of severe hypoxemia as defined above (*P* = 0.003). Plasma HBP was also positively correlated with severity of shock as indicated by dosage of noradrenaline and plasma lactate concentrations at days 1–4 (see Additional file 2: Figure S1 for day 1 data).

### HBP increases in vitro permeability

**Fig. 2:**
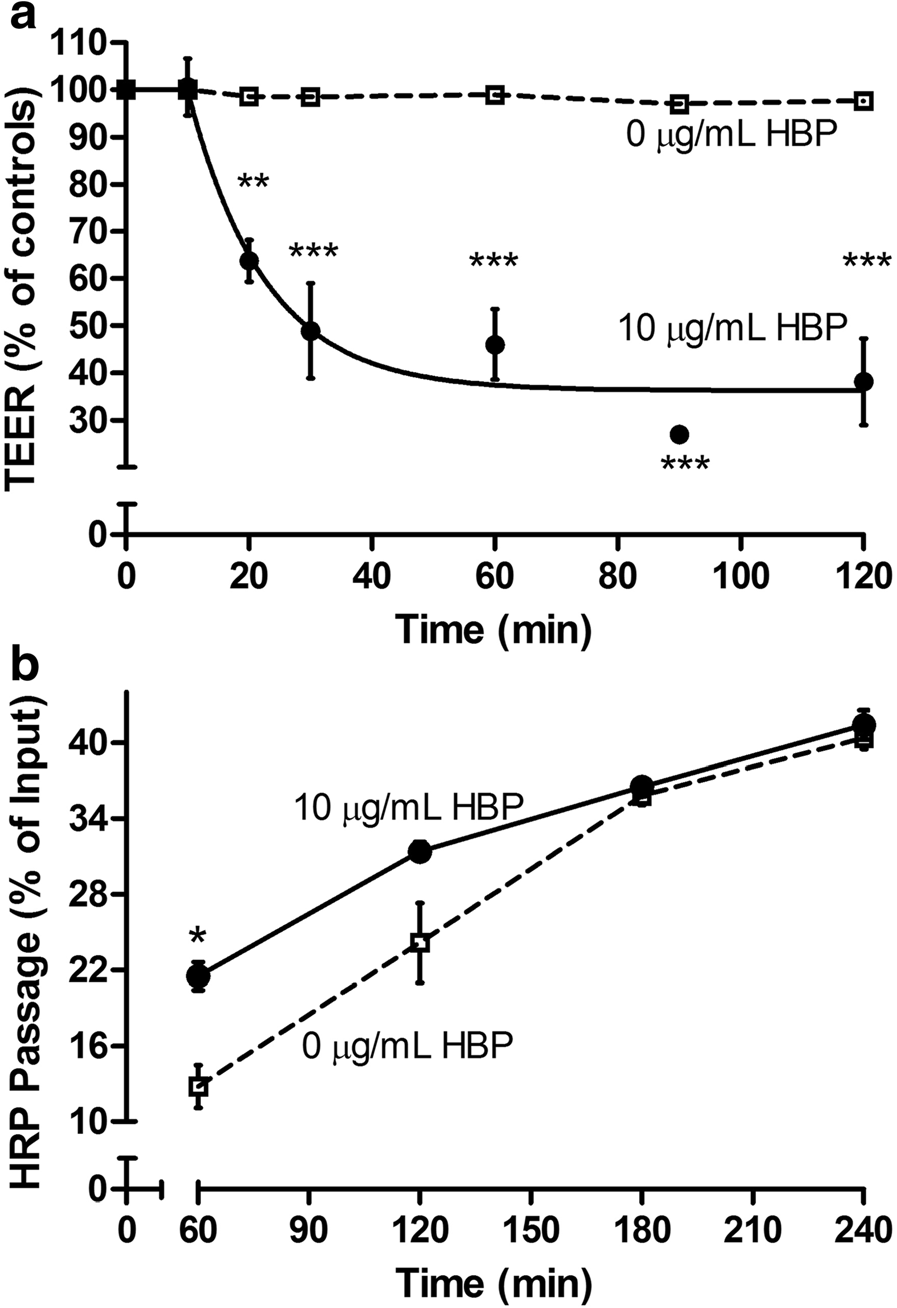
HBP increases the permeability of human endothelial cell monolayers. **a** EA.hy926 cells were grown to confluence on permeable supports and stimulated with HBP. TEER across the filter was monitored over time. The overall difference was determined by two-way ANOVA (treatment effect *P* = 0.001, time effect *P* < 0.001). **b** HRP was added to the top chamber, and HRP passage was monitored over time. Values are normalized to the TEER of empty inserts. The overall difference was determined by two-way repeated measures ANOVA (treatment effect *P* = 0.009, time effect *P* < 0.001). In both experiments, Sidak’s multiple comparison post hoc test was used to compare HBP treatment and control at each time point. *Error bars* are standard error of the mean, *n* = 3. Some error bars are not visible due to scale. *P < 0.05, **P < 0.01, ***P < 0.001

Having established that elevated HBP is associated with physiological findings in keeping with enhanced vascular permeability in septic patients, we went on to further investigate HBP’s mechanisms of action in human endothelial cells. To confirm previous results demonstrating a permeability-increasing effect of HBP on endothelial cell monolayers [2], effects of HBP upon the permeability of endothelial cell monolayers were measured 1.5 h following HBP stimulation. Cell monolayers stimulated with HBP had lower TEER and higher HRP passage than controls indicating that HBP induced increased human endothelial cell permeability (Fig. 2a, b). Cell monolayers stimulated with HBP reached a minimum TEER by 30 min to 1 h following stimulation (Fig. 2a). Similarly, monolayers stimulated with HBP had a higher HRP passage at the earliest time points measured at 1 h, after which HRP diffusion reached equilibrium in all conditions (Fig. 2b).

### HBP acts by binding endothelial proteoglycans

**Fig. 3:**
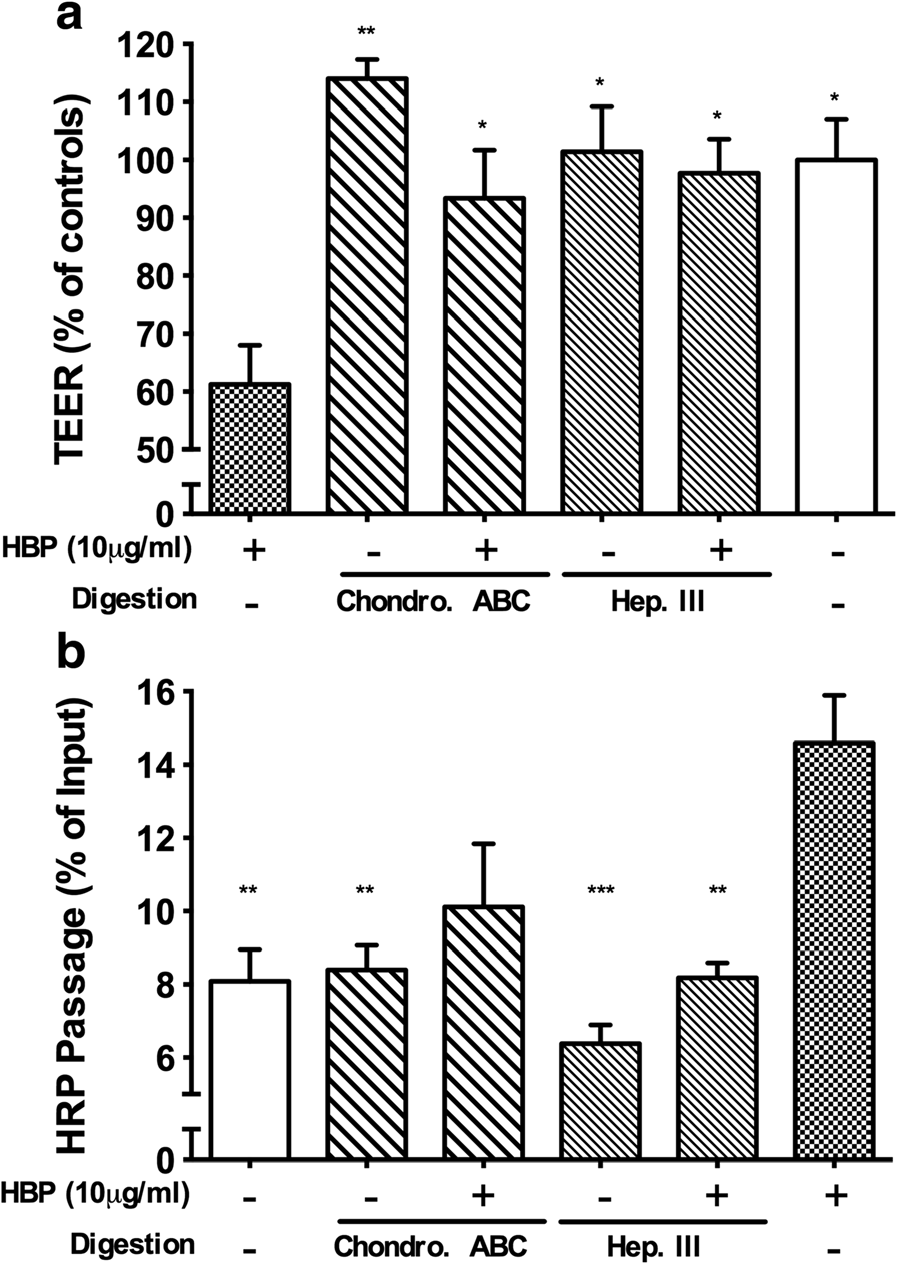
Effects of enzymatic removal of heparan sulfate or chondroitin sulfate by Heparinase III and Chondroitinase ABC on HBP-induced permeability increases. EA.hy926 cells were grown to confluence on permeable supports and treated with heparinase III (Hep. III) or Chondroitinase ABC (Chondro. ABC) for 1 h and then stimulated with HBP. **a** TEER was measured after 1.5 h after HBP stimulation. **b** HRP was also added to the top chamber, and HRP passage was measured 2 h after HBP stimulation. TEER values are normalized to the TEER of empty inserts. *Error bars* are standard error of the mean, *n* = 3 for each condition. In both experiments, one-way ANOVA with Dunnett’s test for multiple comparisons was used to compare each group to the condition with HBP and no enzyme treatment (*far right*). *P < 0.05, **P < 0.01, ***P < 0.001

In order test our hypothesis that binding of HBP to endothelial proteoglycans is required for the increased permeability, Heparinase III or Chondroitinase ABC were used to selectively cleave either heparan sulfate or chondroitin sulfate/dermatan sulfate, respectively, from the endothelial surface [3, 22–24]. Pre-treatment with Heparinase III and Chondroitinase ABC did not affect basal permeability but attenuated HBP-induced increase in permeability suggesting a role for proteoglycans as receptors in HBP-induced vascular endothelial permeability increase (Fig. 3).

### HBP increases permeability through PKC and the Rho-kinase pathways

**Fig. 4:**
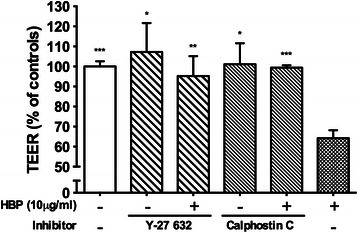
Effect of inhibition of signaling pathways in HBP-induced permeability increases. EA.hy926 cells were grown to confluence on permeable supports and treated with Y-27 632 (Rho-kinase inhibitor) and Calphostin C (PKC inhibitor) for 1 h and then stimulated with HBP. TEER across the filter was measured 1.5 h after HBP stimulation and is normalized to empty inserts. *Error bars* are standard error of the mean, *n* = 3 for each condition. One-way ANOVA with Dunnett’s test for multiple comparisons was used to compare each group to the condition with HBP and no inhibitor (*far right*). *P < 0.05, **P < 0.01, ***P < 0.001

Because activation of the PKC pathway may increase endothelial permeability [25] and because HBP is shown to stimulate PKC activity [26], we tested the hypothesis that HBP increases permeability via the PKC pathway. HBP-induced permeability increase could be blocked by the unselective PKC inhibitor Calphostin C. Activation of PKC is reported to increase permeability of endothelial cells via the Rho-kinase pathway [27]. The Rho-kinase inhibitor Y-27 632 also attenuated HBP-induced increases in permeability (Fig. 4).

### HBP alone induced acute lung injury in vivo

**Fig. 5:**
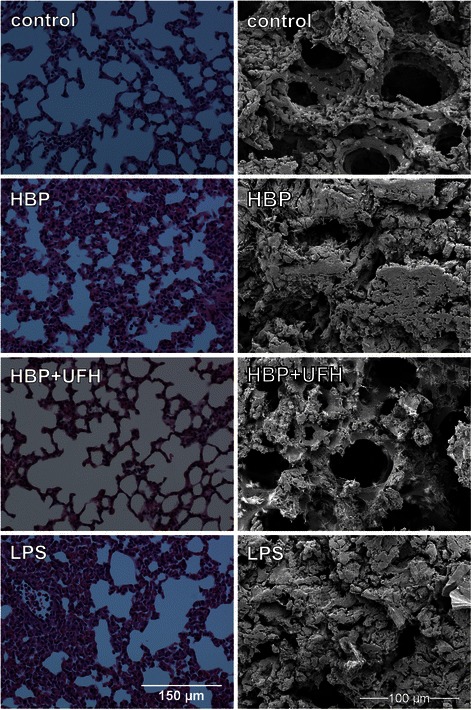
HBP-induced signs of acute lung injury in mice in vivo. Mice were injected with intravenous heparin-binding protein (HBP) and/or unfractionated heparin (UFH) followed by continuous infusion for 1 h. Controls received vehicle (10 mM phosphate-buffered saline). The lungs were stained with hematoxylin and eosin (*left*) or analyzed by scanning electron microscopy (*right*). Hematoxylin and eosin-stained sections were scored for alveolar thickness, capillary congestion, and cellularity (see Table 2). Images from sections with a median overall score are shown. Histologic and electron microscopic images of mice treated with intraperitoneal lipopolysaccharide (LPS) from *Escherichia coli* 0111:B4 in a dose of 0.25 mg for 4 h are presented in lower panels

While HBP is associated with findings suggestive of increased vascular permeability in human septic shock, this association does not prove causality because many other mediators contribute to human septic ARDS. Therefore, we used a murine model to test the hypothesis that HBP causes acute lung injury (ALI) in vivo. HBP administration induced histologic features characteristic of ALI including increased cellularity and overall lung injury score compared to vehicle-treated control animals (Table 2 and Fig. 5). Electron microscopy showed protein deposits and almost complete disappearance of alveoli. HBP induced histological and electron microscopic changes very similar to those observed after LPS administration (Fig. 5). Plasma concentration of HBP at the end of the experiment was 400 ± 157 ng/mL in HBP-treated animals and 1.1 ± 1.5 ng/mL in control animals.

**Table 2:** Lung histologic injury score

	Alveolar thickness	Capillary congestion	Cellularity	Overall score
Vehicle (*n* = 3)	0.33 ± 0.88	0.5 ± 0.05	0.67 ± 0.29	0.5 ± 0.33
HBP (*n* = 3)	1.89 ± 0.84	1.78 ± 0.69	2.06 ± 0.59*	1.91 ± 0.7*
HBP + UFH (*n* = 3)	0.67 ± 1.15	2.67 ± 0.58	0.33 ± 0.58^#^	1.22 ± 0.69
LPS (*n* = 3)	2.5 ± 0.71*	2.25 ± 0.35*	2.00 ± 0.0*	2.25 ± 0.35*

One-way ANOVA. The Sidak method was used to correct for multiple comparisons

*UFH* unfractionated heparin, *LPS* lipopolysaccharide

*P < 0.05 compared to vehicle, ^#^ *P* < 0.05 compared to HBP

### The effect of HBP is inhibited by heparins

**Fig. 6:**
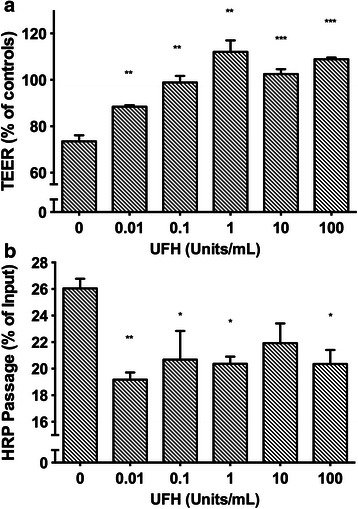
Unfractionated heparin blocked HBP-induced permeability increases. EA.hy926 cells were grown to confluence on permeable supports and stimulated with 10 μg/mL HBP, pre-incubated with the indicated dose of heparin. **a** TEER was measured 1.5 h after stimulation and is normalized to empty inserts. **b** HRP was also added to the top chamber, and HRP passage was measured 2 h after stimulation. *Error bars* are standard error of the mean, *n* = 3 for each condition. One-way ANOVA with Dunnett’s test for multiple comparisons was used to compare each group to the condition with HBP and no heparin (*far left*).*P < 0.05, **P < 0.01, ***P < 0.001

**Fig. 7:**
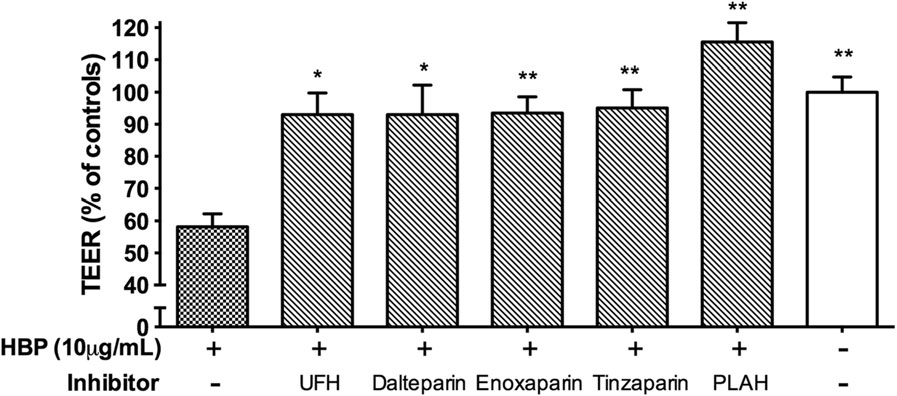
Low molecular weight heparins blocked HBP-induced permeability increases. EA.hy926 cells were grown to confluence on permeable supports and stimulated with 10 μg/mL HBP, pre-incubated with the indicated inhibitor. TEER was measured after 1.5 h after stimulation and is normalized to empty inserts. *Error bars* are standard error of the mean, *n* = 3 for each condition. One-way ANOVA with Dunnett’s test for multiple comparisons was used to compare each group to the condition with HBP and no inhibitor (*far left*). *UFH* unfractionated heparin. *P < 0.05, **P < 0.01

**Fig. 8:**
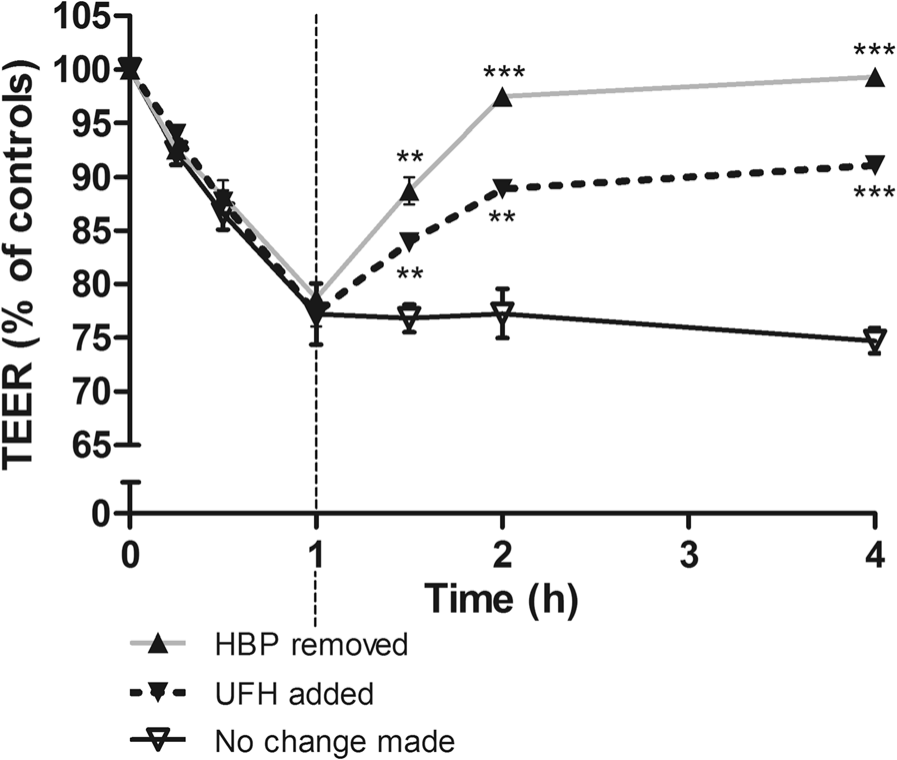
Unfractionated heparin reversed HBP-induced permeability increases. EA.hy926 cells were grown to confluence on permeable supports and stimulated with 10 μg/mL HBP. At 1 h following stimulation, HBP-containing media were removed and replaced with fresh media (*gray line*, treatment 1), or heparin was added to a final concentration of 3 U/mL (*dashed line*, treatment 2), or no change was made (*black line*, treatment 3). TEER across the filter was monitored over time and is normalized to empty inserts. The overall difference was determined by two-way repeated measures ANOVA (treatment effect *P* = 0.002, time effect *P* < 0.001). Sidak’s multiple comparisons post hoc test was used to compare treatments 1 and 2 to treatment 3 at each time point. *Error bars* are standard error of the mean, *n* = 3 for each intervention. *UFH* unfractionated heparin. **P < 0.01, ***P < 0.00

Based on the observation that heparins are known to bind to HBP [28], we hypothesized that heparins could prevent the permeability-increasing effects of HBP. Heparin prevented HBP-induced increased permeability: there was dose-dependent inhibition of altered TEER and HRP permeability following co-stimulation of human endothelial cells with HBP and unfractionated heparin (UFH) (Fig. 6). Maximum inhibition of permeability increases by HBP was seen at concentrations of UFH in the range of 0.1–1 U/mL. Low molecular weight heparins in the therapeutic range also inhibited the TEER-increasing effect of HBP (Fig. 7). In another set of experiments, UFH was added to cells after 1 h of stimulation with HBP. TEER increased to 89 ± 1.0 % of baseline 1 h after UFH was added whereas HBP-treated cells to which no UFH was added remained at 77 ± 4.0 % of baseline (*P* < 0.01, Fig. 8). Treatment with UFH prior to administration of HBP in vivo appeared to prevent both histological and electron microscopic appearance, but no significant change in histologic score could be detected (Fig. 5 and Table 2).

## Discussion

Our results show that increased plasma HBP concentration in human septic shock is associated with increased vascular leak as reflected indirectly by percent fluid overload and the severity of hypoxemia. In addition, HBP increased permeability in vitro. Furthermore, administration of HBP in a murine model rapidly induces a lung injury similar to that observed after LPS administration. The presence of heparan sulfate and chondroitin sulfate moieties on the endothelial cell surface is required for HBP-induced increased permeability as shown by enzymatic degradation of these compounds, which completely abolished the permeability-increasing effect of HBP. Downstream signaling occurs via the protein kinase C and Rho-kinase pathways as shown by experiments using selective inhibitors. The permeability increase by HBP in human endothelial cells was inhibited by pre-treatment and post-treatment with UFH and low molecular weight heparins.

To the best of our knowledge, this is the first report showing that increased plasma HBP levels are associated with the presence or development of severe hypoxemia in a cohort of patients suffering from septic shock. These results are in line with a study showing that increased plasma HBP at admission in trauma patients is correlated with development of ARDS [29]. The results also align with the association between hypoxemia and plasma levels of HBP in a small cohort of patients with influenza infection [30]. However, our results are in contrast with a recent study in which there was no correlation between HBP and development of hypoxemia in patients with severe sepsis [31]. Given the small number of septic patients in that study (*n* = 83), it could be hypothesized that difference in results could be related to a possible false negative due to lower power in the study by Tydén et al. compared to the higher sample size (*n* = 341) and statistical power of our study. The finding that HBP concentrations at baseline in VASST are correlated with indirect markers of increased systemic vascular leakage during the first days of septic shock corroborates previous results suggesting that increased plasma HBP levels at hospital admission predict shock [5–7]. Taken together, these results indicate that plasma levels of HBP may be used to identify patients at risk for development of ARDS and increased systemic vascular leakage and indicate that HBP may be a clinically important mediator of permeability increase in septic shock. The finding that the associations between HBP and markers suggestive of increased permeability were weak could reflect the complex pathophysiology of sepsis with multiple redundant pathways leading to increased permeability [1]. Moreover, indirect markers of increased permeability are likely to be influenced by factors, which are unrelated to changes in permeability. The timing of the plasma sampling may also falsely underestimate the association between HBP and markers of permeability, as discussed below.

Topical application of HBP has previously been show to induce rapid leakage of FITC-labeled dextran in the hamster cheek pouch preparation [2]. However, to date, the effects of intravenous HBP on a whole animal model have not been investigated. Our finding that HBP rapidly induced histological changes consistent with acute lung injury supports the hypothesis that HBP induces increased capillary leak in the lung in vivo and supports a causal relationship between HBP and ARDS.

HBP binds to proteoglycans on endothelial cells, but the functional importance of this binding for increases in permeability has not been investigated before [3, 4]. Proteoglycans are membrane-bound molecules with a protein core to which polysaccharides containing heparan sulfate (HS) and chondroitin sulfate (CS) chains are bound. The syndecans [1–4] are composed of a transmembrane protein core to which HS chains and sometimes CS chains are attached. The glypicans [1–6], with the exception of glypican-5, carry only HS side chains [32, 33]. To date, the expression of all syndecans and glypican-1 and glypican-4 has been described on endothelial cells and syndecan-4 is the predominant syndecan in cultured human endothelial cells [34, 35]. Our result showing that cleavage of both HS and of CS can inhibit the permeability-increasing effect of HBP indicates that syndecans act as receptors for HBP [36]. Proteoglycans are known to act both as primary receptors and as co-receptors that facilitate binding of agonists to other receptors, and thus, the involvement of other receptors for HBP remains a possibility [32, 34].

Our screening of potential intracellular signaling pathways identified PKC as one of the most likely pathways mediating the permeability-increasing effect of HBP on endothelial cells. The result that inhibition of PKC inhibits the permeability-increasing effect of HBP aligns with previous results showing that HBP increases intracellular calcium and activates PKCα in endothelial cells [2, 26]. Interestingly, activation of syndecan-4 has been suggested to influence stress fiber formation in fibroblasts through a calcium-independent PKCα activation mechanism indicating that increased intracellular calcium may not be a prerequisite for increases in permeability [37, 38]. At present, it is unclear if Rho-kinase activation is downstream of PKC activation or if it represents a parallel pathway.

One small high-quality RCT has investigated the effect of heparin treatment on mortality in sepsis and could not demonstrate a benefit of heparin treatment on mortality or severity of ARDS [39]. However, several retrospective analyses and meta-analyses have indicated that heparin treatment may reduce mortality in septic shock [40–42] while one retrospective study could not demonstrate an effect of heparins on ARDS [43]. Given the association between HBP and clinical signs consistent with vascular leak and the permeability-increasing effect in vitro, HBP represents a potential target for therapeutic intervention in sepsis. Our result that both UFH and low molecular weight heparins inhibited permeability increases induced by HBP suggests a cogent rationale for further studies of heparin(s) to prevent sepsis-induced ARDS and vascular leak. Furthermore, our results raise the possibility that HBP levels in plasma may be used to identify a subset of patients with septic shock that may benefit from treatment with heparin in future trials. It should be noted that a recent meta-analysis suggested that safety aspects of heparin in sepsis are underreported, and we conclude that risk of bleeding is a potential concern for the application of heparin in this setting [41].

We acknowledge that this study has several limitations. Firstly, baseline blood samples in the VASST cohort were collected within 2 h of start of treatment with the study drug, which occurred about 12 ± 9 h after meeting inclusion criteria [9]. Given that plasma HBP levels change rapidly [6], we cannot exclude that the variability in the timing of blood sampling could have influenced our results and potentially underestimated the association between HBP and our indirect clinical markers of increased permeability. Secondly, the high cost of recombinant HBP limited the number and length of in vivo experiments that could be performed and prevented us from a more detailed evaluation of physiological effects by HBP in vivo.

## Conclusions

Taken together, our clinical and experimental data suggest a causal relationship between HBP, increased permeability, and ARDS in human sepsis. Unfractionated heparin and low molecular weight heparins are potential drugs to prevent excessive HBP-induced increases in vascular leak in sepsis.

## Additional files

Additional file 1: Online data supplement. (DOCX 104 kb)
Additional file 2: Figure S1. Elevated plasma HBP levels are associated with increased plasma lactate and maximum dose of norepinephrine at day 1 after admission. (A) Scatterplot of plasma HBP levels and maximum dose of norepinephrine on day 1. (B) Scatterplot of plasma HBP levels and plasma lactate concentration at day 1. Dotted lines mark median value for HBP and norepinephrine dose or plasma lactate concentration, respectively. Spearman’s non-parametric correlation coefficient (rho) is given in the figures. (JPEG 886 kb)

## Abbreviations

ALI: Acute lung injury
APACHE: Acute Physiology and Chronic Health Evaluation
ARDS: Adult respiratory distress syndrome
CS: Chondroitin sulfate
DMEM: Dulbecco’s modified Eagle’s medium
FBS: Fetal bovine serum
GAGs: Glycosaminoglycans
HBP: Heparin-binding protein
HRP: Horseradish peroxidase
HS: Heparan sulfate
LMWH: Low molecular weight heparin
LPS: Lipopolysaccharide
PBS: Phosphate-buffered saline
PKC: Protein kinase C
TEER: Trans-endothelial electrical resistance
UFH: Unfractionated heparin
VASST: Vasopressin and Septic Shock Trial
