# Erratum to: Heparin-binding protein is important for vascular leak in sepsis

**DOI:** 10.1186/s40635-017-0119-4

**Published:** 2017-01-20

**Authors:** Peter Bentzer, Jane Fisher, HyeJin Julia Kong, Mattias Mörgelin, John H. Boyd, Keith R. Walley, James A. Russell, Adam Linder

**Affiliations:** 1 Department of Anesthesia and Intensive Care, Helsingborg Hospital, Helsingborg, Sweden; 2 Department of Clinical Sciences Lund, Lund University, Lund, Sweden; 3 Department of Infectious Diseases, University of Lund and Skåne University Hospital, Getingevägen, Lund, SE-221 85 Sweden; 4 Centre for Heart Lung Innovation, Division of Critical Care Medicine, St. Paul’s Hospital, University of British Columbia, Vancouver, BC Canada

## Erratum

The original article [1] contains an oversight whereby Fig. 7 presents data on a molecule, ‘PLAH’ that was intended to be omitted from this article.

The reason for the intended omission of this molecule throughout the article was due to complications in obtaining the license for both the use of and presentation of research involving this molecule, and its presence within Fig. 7 was unintentional.

As such, the appropriate version of Fig. 7 can be seen below.

**Fig. 7:**
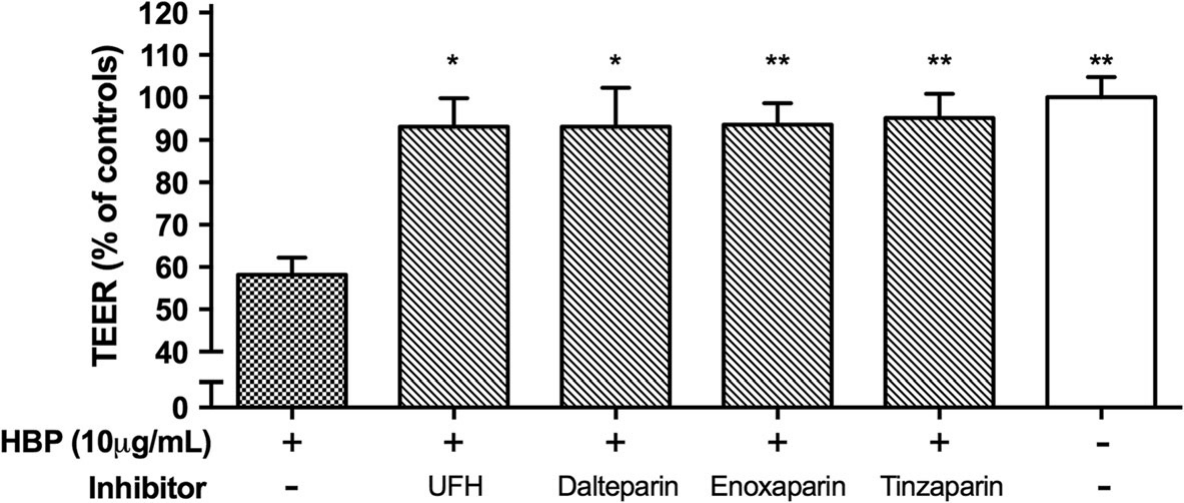
Low molecular weight heparins blocked HBP-induced permeability increases. EA.hy926 cells were grown to confluence on permeable supports and stimulated with 10 μg/mL HBP, pre-incubated with the indicated inhibitor. TEER was measured after 1.5 h after stimulation and is normalized to empty inserts. *Error bars* are standard error of the mean, *n* = 3 for each condition. One-way ANOVA with Dunnett’s test for multiple comparisons was used to compare each group to the condition with HBP and no inhibitor (*far left*). *UFH* unfractionated heparin. *P < 0.05, **P < 0.01
