# Supplementary figures and images for: Heparin-binding protein is important for vascular leak in sepsis
Source: Intensive Care Med Exp. 2016 Oct 4;4:33. doi: 10.1186/s40635-016-0104-3 (PMC5050173; doi:10.1186/s40635-016-0104-3)

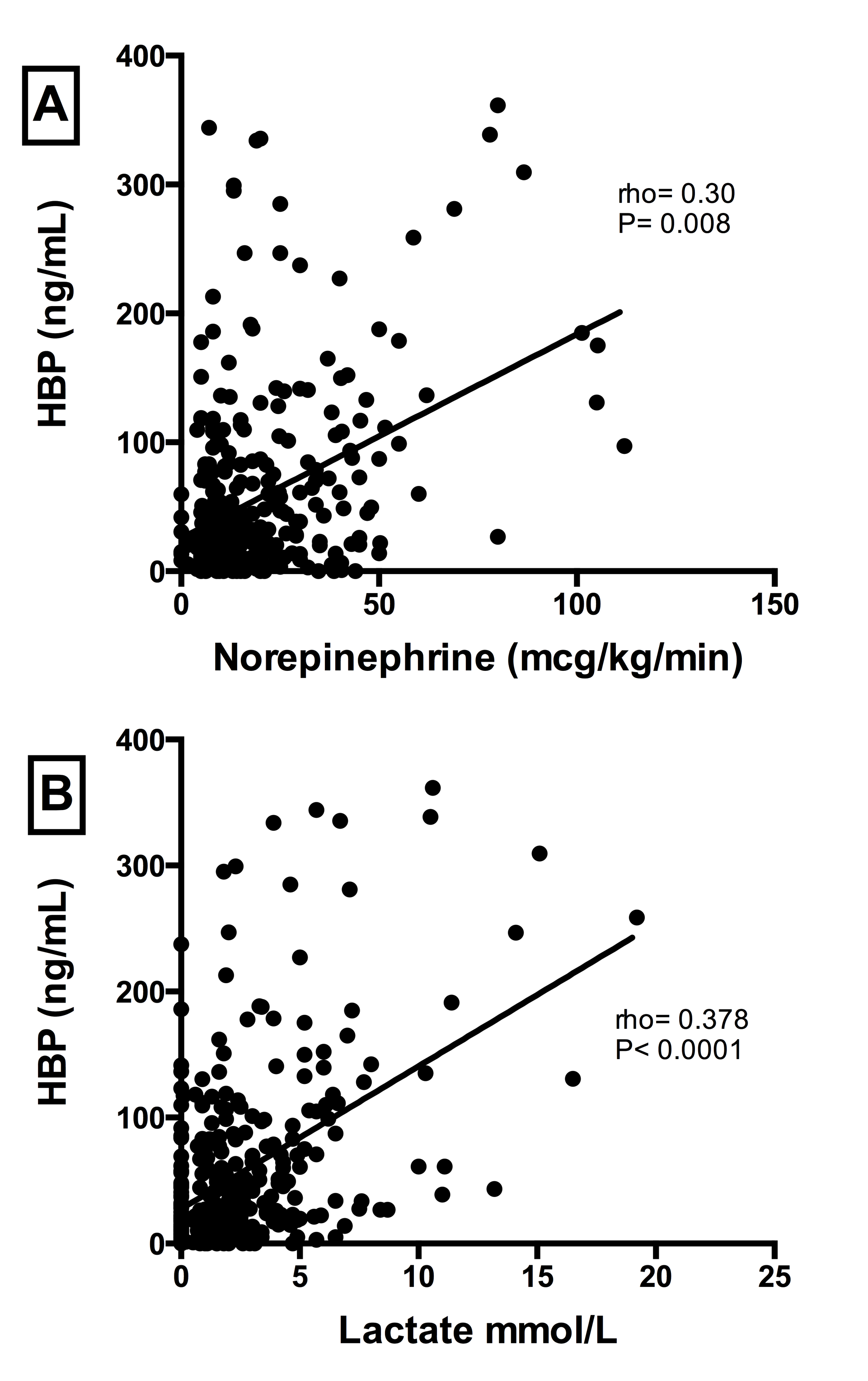

Supplement: Additional file 1: — Online data supplement. (DOCX 104 kb) [file 40635_2016_104_MOESM2_ESM.jpg]
