# Supplementary material for: Heparin-binding protein is important for vascular leak in sepsis
Source: Intensive Care Med Exp. 2016 Oct 4;4:33. doi: 10.1186/s40635-016-0104-3 (PMC5050173; doi:10.1186/s40635-016-0104-3)
Supplement: Additional file 2: Figure S1. — Elevated plasma HBP levels are associated with increased plasma lactate and maximum dose of norepinephrine at day 1 after admission. (A) Scatterplot of plasma HBP levels and maximum dose of norepinephrine on day 1. (B) Scatterplot of plasma HBP levels and plasma lactate concentration at day 1. Dotted lines mark median value for HBP and norepinephrine dose or plasma lactate concentration, respectively. Spearman’s non-parametric correlation coefficient (rho) is given in the figures. (JPEG 886 kb) [file 40635_2016_104_MOESM1_ESM.docx]

**Online data supplement**

**Materials and Methods.**

***In vitro experiments***

*Streptavidin-conjugated horseradish peroxidase (HRP) permeability assay*

Measurement of macromolecular permeability using HRP was performed as described previously (van der Heijden et al., 2011). Cells were stimulated with HBP and/or inhibitors with HRP added to the top chamber. Samples from the bottom chamber of each well were removed. A 1:1 mixture of stabilized peroxide (color reagent A) and stabilized tetramethylbenzidine (color reagent B) (R&D Systems) was added to each sample and incubated for 5 minutes at room temperature. 2N sulfuric acid (R&D Systems) was added to stop the reaction. The absorbance of each well at 450nm was determined using a microtiter plate reader (Tecan Group Ltd). HRP activity in each sample was calculated from a standard curve. All samples were assayed in duplicate. HRP passage is reported as a percentage of the initial HRP input.

***In vivo* experiments.**

*Anesthesia and preparation*

Anesthesia was induced with 4% isoflurane (Schering-Plough Animal Health) in a closed container and maintained with isoflurane via a mask until a tracheostomy was performed. Animals were mechanically ventilated (Ugo Basile Animal Ventilators) to an end-tidal CO_2_ concentration of 4.5 - 6 kPa using volume-controlled mode (tidal volume 6 ml/kg) and a positive end-expiratory pressure of 5 cmH_2_O. Body temperature, measured rectally, was maintained at 37°C. The left femoral artery was cannulated for measurement of arterial pressure and blood sampling. Following cannulation of the internal jugular vein a 6% dextran solution (Meda) at a dose of 3.6 uL/g was administered during 20 min. A maintenance infusion of Ringer´s Acetate was then started at a rate of 0.3 uL/min. Blood gases were analyzed prior to start of infusion of drug or vehicle and after one hour (I-STAT; Abbot Point of Care Inc).

*Histology and electron microscopy of lung*

Tissue samples for histology were fixed in buffered 4% formalin (pH 7.4) at room temperature for 7 days after which the samples were transferred to 70% ethanol. Following dehydration samples were imbedded in paraffin (Histolab Products AB), cut into 4 µm sections, and mounted. After removal of the paraffin, tissues were stained with Mayers hematoxylin (Histolab Products AB) and eosin (Surgipath Medical Industries, Inc.). Tissue samples for electron microscopy were fixed in 0.15 sodium cacodylate, 2.5% glutaraldehyde, pH 7.4. After fixation, samples were washed, dehydrated, critical point dried, and sputtered with palladium/gold as described in more detail earlier (Herwald et al., 2003). Specimens were examined in a Philips/FEI XL-30 fiel emission scanning electron microscope (JEOL) operated at an acceleration voltage of 5 kV, working distance of 7,5 mm and a magnification of 500 – 5000 times.

**References**

E 1. van der Heijden M, van Nieuw Amerongen GP, van Bezu J, Paul MA, Groeneveld AB, van Hinsbergh VW: Opposing effects of the angiopoietins on the thrombin-induced permeability of human pulmonary microvascular endothelial cells. PloS One 2011; 6:e23448.

E 2. Herwald H, Cramer H, Mörgelin M, Russell W, Sollenberg U, Norrby-Teglund A, Flodgaard H, Lindbom L, Björck L. M protein, a classical bacterial virulence determinant, forms complexes with fibrinogen that induce vascular leakage. Cell. 2004; 116: 367-79.
